# Exploring the taxonomic composition of two fungal communities on the Swedish west coast through metabarcoding

**DOI:** 10.3897/BDJ.7.e35332

**Published:** 2019-09-04

**Authors:** Alice Retter, R. Henrik Nilsson, Sarah J. Bourlat

**Affiliations:** 1 University of Vienna, Vienna, Austria University of Vienna Vienna Austria; 2 University of Gothenburg, Göteborg, Sweden University of Gothenburg Göteborg Sweden; 3 Gothenburg Global Biodiversity Centre, Gothenburg, Sweden Gothenburg Global Biodiversity Centre Gothenburg Sweden; 4 Zoological Research Museum Alexander Koenig, Bonn, Germany Zoological Research Museum Alexander Koenig Bonn Germany

**Keywords:** Biodiversity, Ecology, Marine fungi, Metabarcoding, High-throughput sequencing, Fungal diversity

## Abstract

**Background:**

Fungi are heterotrophic, unicellular or filamentous organisms that exhibit a wide range of different lifestyles as, e.g., symbionts, parasites, and saprotrophs. Mycologists have traditionally considered fungi to be a nearly exclusively terrestrial group of organisms, but it is now known that fungi have a significant presence in aquatic environments as well. We know little about most fungi in limnic and marine systems, including aspects of their taxonomy, ecology, and geographic distribution. The present study seeks to improve our knowledge of fungi in the marine environment. The fungal communities of two coastal marine environments of the Kattegat sea, Sweden, were explored with metabarcoding techniques using the nuclear ribosomal internal transcribed spacer 2 (ITS2) metabarcode. Our data add new information to the current picture of fungal community composition in benthic and coastal habitats in Northern Europe.

**New information:**

The dataset describes the number of operational taxonomic units (OTUs) and their taxonomic affiliations in two littoral gradients sampled on the Swedish west coast, Gothenburg municipality. Our data include basic diversity indices as well as chemical and edaphic sediment/soil parameters of the sampling sites. From the sites, 3470 and 4315 fungal OTUs, respectively, were recovered. The number of reads were 673,711 and 779,899, respectively, after quality filtering. Within the benthic sites, more than 80% of the sequences could not be classified taxonomically. The phylum composition of the classifiable sequences was dominated in both localities by Dikarya, which made up around 33% of the OTUs. Within Dikarya, Ascomycota was the dominant phylum. Guild assignment failed for more than half of the classifiable OTUs, with undefined saprotrophs being the most common resolved guild. This guild classification was slightly more common in the ocean sediment samples than in the terrestrial ones. Our metadata indicated that ocean sites contain organisms at a lower trophic level and that there are predominantly endophytic, parasitic, and pathogenic fungi in the marine environments. This hints at the presence of interesting and currently poorly understood fungus-driven ecological processes. It is also clear from our results that a very large number of marine fungi are in urgent need of taxonomic study and formal description.

## Introduction

The fungal kingdom may hold as many as 3.8 million extant species, but only around 146,000 have been formally described so far (Hawksworth and Lücking 2017, Kirk 2019). Many fungi are inconspicuous with largely cryptic lifestyles, and many species from across the fungal tree of life do not seem to form conspicuous morphological structures such a fruiting bodies in any way that is currently appreciated (Tedersoo et al. 2017). Fungi take up dissolved carbonic matter by absorption and they interact with all major groups of organisms (Taylor et al. 2014); indeed, the fungal kingdom comprises organisms with a wide range of ecologically important lifestyles, including decomposers, parasites, pathogens, and symbionts/mutualists (Stajich et al. 2009). Fungi are traditionally thought of as terrestrial organisms, and the relatively few aquatic taxa known and studied in the pre-molecular era were largely associated with submerged terrestrial substrates such as driftwood and plant debris. Molecular studies have changed that by showing that fungi permeate not only terrestrial but also aquatic systems (Amend et al. 2019, Gladfelter et al. 2019). For instance, we now know that fungi are found throughout marine systems, from the sediment through the water column to various associations with marine animals, plants, and algae (Jones and Pang 2012, Gnavi et al. 2014, Raghukumar 2017).

Although we are nowhere near a complete understanding of the ecological roles and taxonomic diversity of fungi in aquatic systems, data are starting to emerge. Recent results suggest that fungi degrade significant amounts of biomass in aquatic systems, just like they do on land, where biomass is degraded by fungal saprotrophs into simpler biomolecules that are subsequently absorbed (Raghukumar 2017). There is also evidence that the zoospores of chytrids are grazed by marine zooplankton, which has an impact on higher-level consumers in the marine trophic chain (Lepelletier et al. 2014). Other marine fungi are parasitic or pathogenic, thereby restructuring aquatic nutrient flows through processes such as the mycoloop (Kagami et al. 2014, Grossart et al. 2019). Fungi are, in other words, potentially able to structure and control the functioning of aquatic food webs (Jones 2011, Grossart et al. 2016).

Studying marine fungi through traditional lab and culture-based methods is a very slow and painstaking process, as many fungi are difficult to raise in culture (Stajich et al. 2009). This is particularly true for fungi that form associations with other organisms, such as endosymbionts and endoparasites. The marine environment adds another layer of complexity to cultivation efforts through aspects such as salt levels, pH, temperature, and light regimes. As an alternative to cultivation, environmental DNA of fungal communities is increasingly assessed by metabarcoding, a biodiversity assessment method that combines DNA-based identification and high-throughput (HTS) DNA sequencing (Nilsson et al. 2019a). Metabarcoding can be used to characterise entire fungal communities using a relatively short DNA barcode, such as the internal transcribed spacer region (ITS) of the nuclear ribosomal DNA operon (Schoch et al. 2012). Barcode amplification relies on taxon-specific primers that ideally target all fungi. Ill-chosen primers will lead to biases in terms of what taxa are recovered, masking environmental signals and distorting the view of the fungal communities obtained (Ihrmark et al. 2012, Tedersoo et al. 2015). What species and what taxonomic levels are recovered is also dependent on the completeness of the reference databases for fungi, the most complete and up-to-date one being UNITE (Nilsson et al. 2019b).

Numerous studies over the last decade have sought to assess the fungal biodiversity in different local areas and habitats (Hiiesalu et al. 2014, Urbina et al. 2016, Pec et al. 2017) as well as globally (Tedersoo et al. 2014, Davison et al. 2015). A fair number of studies have examined terrestrial fungal diversity in Sweden (Lindahl et al. 2007, Ihrmark et al. 2012, Varenius et al. 2016), but studies of marine fungi in Sweden are much more rare (Eriksson 2014, Tibell 2016, Tibell et al. 2019). The purpose of this study was to investigate how well fungal taxa collected from coastal sediments of the Kattegat sea in western Sweden have been documented so far. As in many other areas, waters in the Kattegat sea have been impacted by anthropogenic pressures such as eutrophication and fisheries (Josefson et al. 2018). To be able to track future changes in fungal diversity and abundance, it is important to generate baseline datasets. With this dataset, we seek to add to the taxonomic and ecological knowledge of marine fungal communities through ITS2-based metabarcoding of two coastal localities on the Swedish west coast.

## Project description

### Study area description

**Figure 1.**
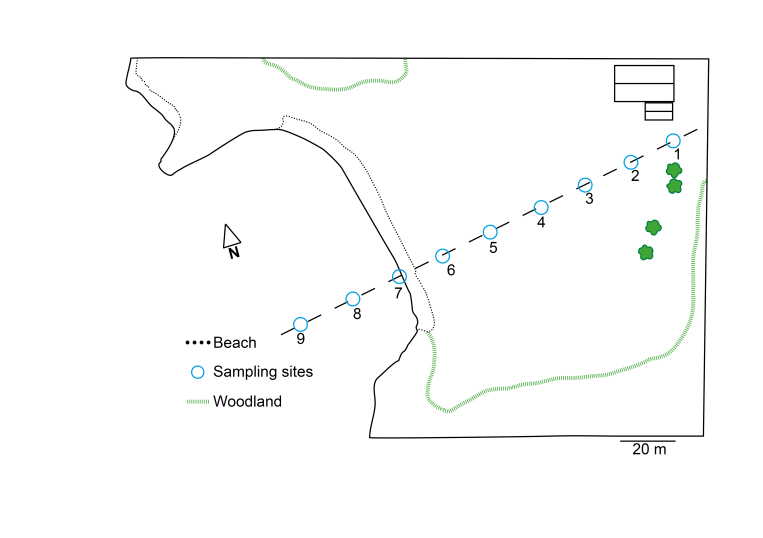
Map of the sampled transect at Askimsbadet.

**Figure 2.**
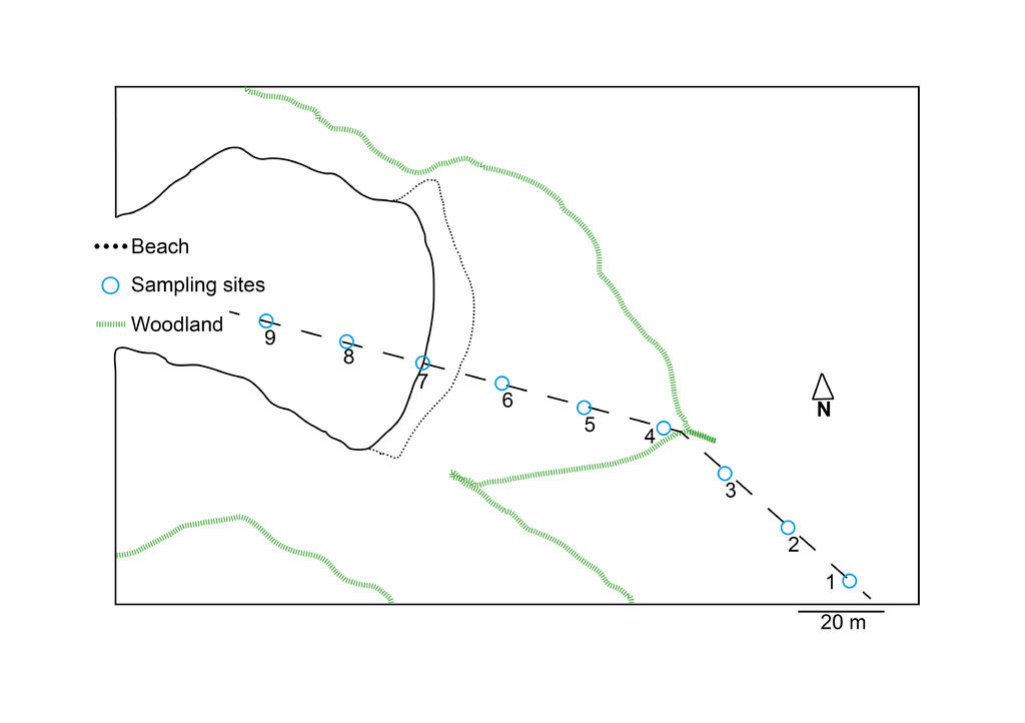
Map of the sampled transect at Stora Amundön.

We targeted two different localities of similar ecology and habitat. The first sampling was carried out at Askimsbadet, Gothenburg Municipality, Västra Götalands county (Fig. 1). This locality is characterized by a shallow saltwater bay with a short intertidal shoreline and a sandy beach becoming meadow-like with high grass and reeds. The second sampling was undertaken on the island Stora Amundön, Gothenburg Municipality, Västra Götalands county (Fig. 2). It consisted of a shallow sandy cove turning into a grassy meadow landscape. The grassland was surrounded by rocks and shrubs and was replaced by mixed woodland after a stretch of around 60 m. Both transects were approximately 160 m long. The geographic coordinates and altitude of each sampling area were recorded, and the ectomycorrhizal plant cover at each site was noted. Sampling sites are numbered according to Figs 1, 2 and are indicated by parentheses in the following plant cover description. The vegetation at the Askimsbadet site consisted of mostly dried seaweed at the shoreline, but gradually turned into a natural reed and grass landscape after around 3 m, with a few grazing plants such as *Lysimachia maritima* and *Argentina anserina* (7). These plants, along with *Rumex* sp. and *Anthriscus sylvestris*, could also be found further up in the meadow landscape (6, 5). They were later replaced by *Taraxacum* sp., *Cirsium* sp. (4, 1), and *Urtica* sp. (3), although *Anthriscus sylvestris* and *Rumex* sp. could be found all along the gradient. The furthest up, genera and species such as *Equisetum* sp., *Taxus* sp., *Thuja* sp., *Malus* sp., *Rubus* sp., and *Calystegia sepium* could be spotted (1). The vegetation at the Stora Amundön site consisted of dried seaweed at the shoreline (7) that was replaced by a mown grass landscape (6, 5, 4) where *Erica* sp., *Taraxacum* sp., *Juniperus* sp. (5), and young *Quercus* sp. (4) could be found. Further up, the landscape transformed into a mixture of deciduous and coniferous forest that consisted of *Quercus* sp., *Juniperus* sp., *Populus* sp., *Betula* sp., *Pinus* sp. (1, 2, 3), and some fern taxa such as *Pteridium* sp. (3) and *Polypodium vulgare* (2).

## Sampling methods

### Sampling description

We followed the soil sampling protocol of Tedersoo et al. (2014) with some slight adaptations to assess a spatial gradient from land through an intertidal shoreline into the ocean. Additionally, pH and salinity were measured at all sampling points. At each of the two localities, soil was collected along the transect, which was sampled every 20 meters, resulting in nine different sampling sites (Figs 1, 2). Of those nine sites, six were located within the meadow or forest stretch, respectively, and were approximately at sea-level, one site was located on the shoreline, and two were sampled from the seabed at approximately 1 to 2 m below the ocean surface. For both transects, the following procedure was adopted. Two samples were taken from each site and diluted 1:1 with freshwater to measure the pH and conductivity of the soil/sediment. The average of these two measurements was used as an estimate of the true values. At each site, 10 replicate cores were sampled with a metal bulb planter, all within a radius of 20 m. Each core consisted of the top 5 cm of soil/sediment and had a diameter of 5 cm. Where applicable, loose litter was removed from the surface of the sampling points. The sample itself thus consisted of the organic and upper mineral layer when sampled from land and the sandy to clay mineral layer when sampled from the ocean floor sediment. From the soil cores obtained, a subset of soil from all sides of the core was picked and placed in a clean plastic tray. The remainder of the soil core was discarded. Within 4 hours of collection, samples were rid of coarse roots and stones and air-dried in a drying room at room temperature. When dry, the samples were placed in Ziploc bags with silica gel and placed in a dark, dry room for storage and subsequent analysis. Subsamples were sent for analysis of soil isotopes and mass fraction of carbon and nitrogen with an isotope ratio mass spectrometer (DeltaV, Thermo Fisher Scientific, Bremen, Germany).

### Quality control

Between each round of sampling, the bulb planter was cleaned with water to avoid carryover of DNA between sampling rounds.

## Geographic coverage

### Description

Both localities are situated in the coastal region of Gothenburg, Sweden and each locality covered an estimated study area of around 2400 m^2^.

### Coordinates

57.591490° and 57.621117° Latitude; 11.903327° and 11.930502° Longitude.

## Temporal coverage

### Notes

Sampling was carried out at the beginning of September 2017.

## Usage rights

### Use license

Creative Commons Public Domain Waiver (CC-Zero)

## Data resources

### Data package title

Exploring the taxonomic composition of two fungal communities on the Swedish west coast through metabarcoding.

### Resource link

https://trace.ncbi.nlm.nih.gov/Traces/sra/sra.cgi?study=SRP189972

### Number of data sets

1

### Data set 1.

#### Data set name

ITS2 metabarcoding of fungi

#### Number of columns

11

#### Description

ITS2 raw data and reads. The data underpinning the analysis reported in this paper are deposited at the GenBank SRA (Kodama et al. 2011) under BioProject number PRJNA530104.

**Data set 1.** 

Column label	Column description
RUN	Accession number (Contains instrument and library information. Is a manifest of data file(s) that are derived from sequencing a library described by the associated EXPERIMENT)
BIO SAMPLE	Accession number (Bio sample is a record of a biological isolate with unique physical properties)
SAMPLE NAME	Each sample name must be unique
EXPERIMENT	Accession number (Each experiment is a unique sequencing result for a specific sample)
MBASES	Total bases
ORGANISM	The source of the sample
ELEV	Elevation
ENV LOCAL SCALE	The local environment from which the sample was taken.
ENV MEDIUM	The medium from which the sample was taken
GEO LOC NAME	The geographical location from where the sample was taken
LAT LON	Latitude and longitude of sampling location

## Additional information

### DNA extraction and ITS2 amplification

To prepare the samples for DNA extraction, the Ziploc bags containing dried soil were rubbed vigorously between both hands for 3 minutes until the soil was transformed into fine dust following the protocol of Tedersoo et al. (2014). From this fine dust, 300 g were subsampled for later DNA extraction. For total DNA isolation we used the DNeasy PowerSoil Kit (Qiagen, Hilden, Germany), following the manufacturer’s instructions. The kit is intended to be used for environmental samples with high humic acid content and other difficult soil types such as sediment. The DNA concentrations after extraction of all samples were measured with a Qubit 3.0 Fluorometer (Thermo Fisher Scientific, Waltham, Massachusetts, USA) and were found to range from a few ng/μl up to ~ 180 ng/μl.

We measured DNA purity with a NanoDrop (Thermo Fisher Scientific, Waltham, Massachusetts, USA) instrument prior to PCR, and sample concentration was quantified with a Qubit 3.0 and the iQuant™ Quantitation Kit. Multiplexed amplicon libraries were constructed according to the two-step PCR protocol described in Bourlat et al. (2016). This method consists of a dual PCR amplification. The first PCR uses amplicon-specific primers including an Illumina adapter overhang (amplicon PCR), and the second, cycle-limited PCR is used for incorporation of Illumina index adapters for multiplexing (index PCR) (Bourlat et al. 2016).

The ITS2 primers (Table 1) used in this study were designed and previously used for the detection of both terrestrial and aquatic fungi (Tedersoo et al. 2015, Wurzbacher et al. 2017). They contain a Nextera-Illumina-Adapter overhang, a mismatch spacer, and the marker-specific sequence. Small letters within the primer sequence represent modified nucleotides, so called PTOs (phosphothioate oligonucleotides). PTOs prevent mismatch corrections by the proofreading polymerase. The forward primer is degenerate and an equimolar mix of the reverse primers is used to improve taxonomic coverage of the fungal kingdom.

Amplicon PCR was conducted with the KAPA HiFi HotStart ReadyMix kit (Roche, Basel, Switzerland), containing an engineered B-family DNA polymerase for fast and versatile high-fidelity PCR that reduces the number of nucleotide incorporation errors produced during PCR amplification (Lindahl et al. 2013). Primers were diluted to a final concentration of 20 pmol/μl, and the reverse primers (ITS4-cwmix1 and ITS4-cwmix2) were mixed at equimolar concentration.

The DNA template concentration of all samples was between 20 and 50 ng/μl. The PCR cocktail of 25 μl reaction volume comprised 12.5 μl KAPA HiFi HotStart ReadyMix (Roche, Basel, Switzerland), 1 μl of forward primer and 1 μl of reverse primer mix at 20 pmol/μl, 9.5 μl of Nuclease-Free Water (Qiagen, Hilden, Germany), and 1 μl of template DNA (20–50 ng/μl). We additionally used a negative (no template DNA) control. Three replicate reactions were carried out for each sample with the following program on a MyCycler™ thermal cycler (Bio-Rad, Hercules, California, USA): initial denaturation for 3 min at 95°C followed by 30 cycles of 30 sec at 95°C, 30 sec at 57°C, 1.5 min at 70°C, and a final elongation cycle for 5 min at 72°C. We kept the number of PCR cycles as low as possible in order to reduce PCR incorporation error and to avoid reaching a limiting stage due to decreased accessibility of template DNA or PCR mixture components (Opel et al. 2010). To check for amplification products, as well as their size and concentration, we ran 3 μl of all PCR replicates on a 2% agarose gel using 1×TAE Buffer and GelRed® (Biotium, California, USA) for DNA staining. We ran the gel at 100 V for up to 45 min. We also verified the individual fragment size with a TapeStation (Agilent Technologies, Santa Clara, California, USA), using the genomic DNA ScreenTape assay. On both the gel and the TapeStation, the fragments were found to have a size of 300–550 bases, which is the expected size of the amplicons generated with our primers. The variability in fragment size is due to ITS2 itself, as its length can vary within different groups of fungi (Tedersoo et al. 2015). The products of all three PCR replicates were pooled and quantified with a Qubit 3.0 Fluorometer (Thermo Fisher Scientific, Waltham, Massachusetts, USA). Pooled products were then sent to Macrogen (Seoul, Korea) for indexing and Illumina MiSeq sequencing (San Diego, California, USA), providing 1.3 Gb of raw data as paired-end reads of 2 × 300 bp.

### Bioinformatics

To analyse community composition and assign taxonomic affiliations to the amplicon sequences, we used the software pipeline micca (Albanese et al. 2015) v. 1.6 that utilizes the third-party software tools Cutadapt (Martin 2011) for primer trimming and VSEARCH (Rognes et al. 2016) v. 2.7.1 for merging, filtering, OTU picking, and taxonomic classification of sequences. We analysed the transects separately to maintain integrity in the subsequent analysis. For each gradient, we assembled the paired-end reads of the adapter trimmed data, requiring a minimal overlap of 50 bases and strict assessment with zero mismatches. Paired reads that did not contain both forward and reverse primers were discarded, and primer sequences were then removed from the merged reads. We filtered all remaining sequences by discarding those that were shorter than 260 bases, and/or had an expected error rate of more than 0.5%. OTU clustering was carried out using a complete-linkage clustering algorithm for denovo greedy clustering. Sequence reads were assigned to operational taxonomic units (OTUs; Blaxter et al. 2005) using the denovo greedy clustering approach of VSEARCH and a sequence similarity cut-off of 97%. Chimeric OTUs, which are artificial sequences formed when two or more biological sequences are joined together and amplified during PCR, were removed using the default approach in micca. Also, singleton (one-sequence) OTUs were excluded from further analyses, as they most probably are the product of PCR/sequencing errors and would thus lead to inflated diversity estimates in the downstream statistical analyses. Taxonomic assignment was done using the VSEARCH-based consensus classifier of micca utilizing the UNITE database reference release, using default parameters (version 7.2, doi 10.15156/BIO/587481; Nilsson et al. 2019b).

We made an effort to manually scrutinize the OTU tables for large OTUs of non-fungal origin, as we wanted our statistics to be based primarily on the fungal component of the microbiomes of these sites. OTU tables of both gradients were therefore checked for non-fungal and ambiguous taxonomic assignments. In addition, the 50 most abundant OTUs of each gradient were manually checked using BLAST in GenBank (https://blast.ncbi.nlm.nih.gov/Blast.cgi) following the guidelines of Nilsson et al. (2012). From the initial 4355 OTUs in the OTU table of Askimsbadet, and the 3510 OTUs in Stora Amundön, each sampling locality saw the exclusion of 40 OTUs. Most of them were found to represent either animals or plants, and a few other OTUs were deemed too ambiguous to be included. We then used the modified OTU table and taxonomic assignments to compute relative abundance tables of sequences at different taxonomic levels. To account for differences in sequencing depth, rarefaction of the OTU tables was done in micca.

### Statistical analysis

For statistical analysis of the gradients, we used the vegan package v.2.4-6 (Oksanen et al. 2012) in R v.3.4.4 (R Core Team 2014). Rarefied and non-rarefied OTU tables from micca were loaded into R, as were the taxonomy data. We then assessed species diversity and computed the rarefaction curves of the gradients, which indicated the extent to which we had managed to sample the entire communities in the gradients. We computed each site’s richness from the non-rarefied OTU tables and calculated the Shannon index from the rarefied OTU tables. The Shannon index gives an estimate of the species composition, which is the effective species number at a given site (alpha diversity; Tuomisto 2010, Jurasinski and Koch 2011). We further used the Shannon index to calculate the gradients' Pielou's evenness, which gives an estimate of the evenness of the distribution of species. Chao1 estimates were calculated from the rarefied OTU tables. These estimates take undiscovered rare species into account and are thus also described as the estimate of the true species richness (Chao 1984). To infer a network plot of similar sites, we computed the distances between sites using the Jaccard dissimilarity index applying the vegan function "vegdist", using the method argument "jaccard". The Jaccard index was calculated from the presence/absence-transformed rarefied OTU tables.

### Results

**Physicochemical properties**: Gradients from both localities show overall similar physicochemical soil properties (Table 2). Exceptions are the total carbon and nitrogen contents of the forest sites in Stora Amundön, which are higher than the others, and the d^15^N ratios, which are close to zero. The shoreline site in Stora Amundön stands out, as the total carbon and nitrogen contents are lower than the average. Moreover, the ^13^C ratio of the ocean site SA9 stands out as lower than what one would expect when comparing with the ocean sites of the other locality, and its C/N ratio is as high as in the forest sites. Conductivity is directly related to the concentration of salt ions in the soil and reaches a few mS in the seabed sediments. The forest sediments in Stora Amundön are more acidic than the meadow and ocean sites and all benthic sites areslightly alkaline.

**Amplicon data**: Between merging paired-end sequences and primer trimming, almost 99% of the sequences were retained (Table 3). A loss in sequences was observed after quality filtering and discarding reads of fewer than 260 bases, which left us with around 97% of the original primer-trimmed sequences. This means a loss of around 13,000 sequences in the Stora Amundön transect and 8900 sequences in the Askimsbadet transect. Only a minor fraction (around 0.08%) of our sequences were identified as chimeric. The statistics of the raw data can be found in Suppl. material 2.

**Figure 3.**
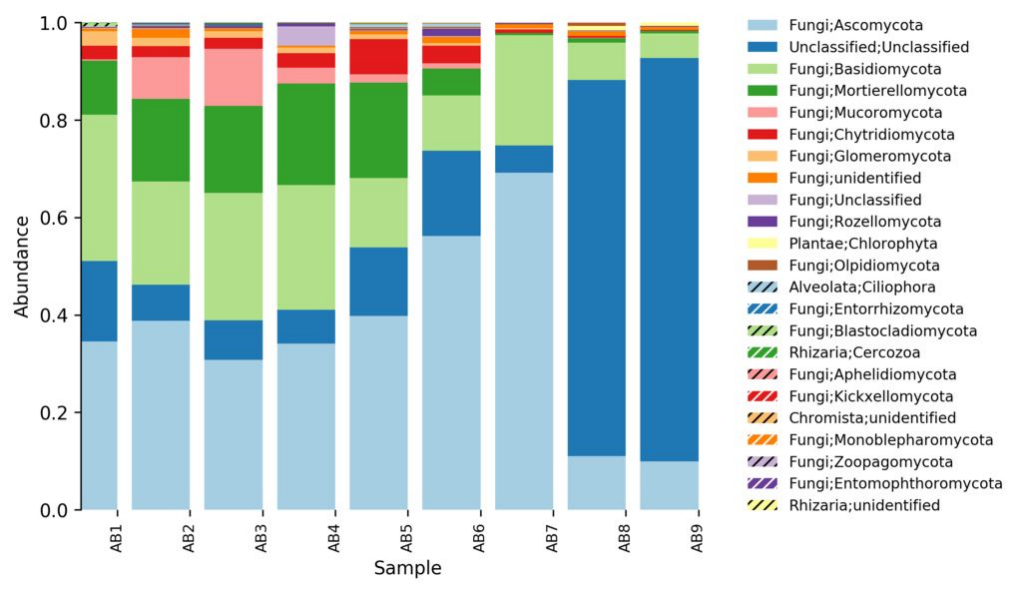
Relative abundance of reads at phylum level (Askimsbadet).

**Figure 4.**
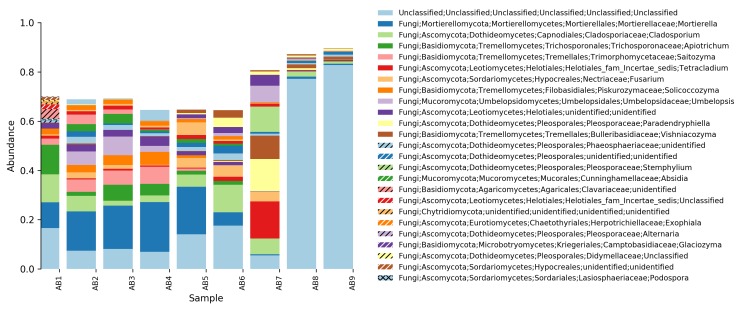
Relative abundance of reads at genus level (Askimsbadet).

**Figure 5.**
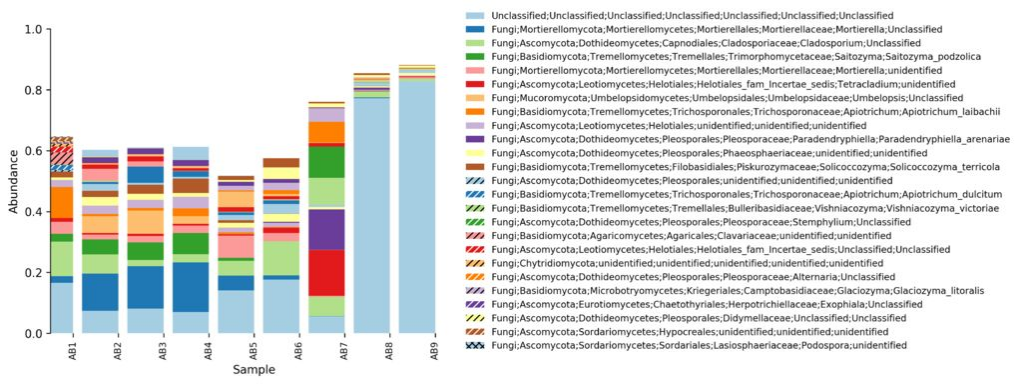
Relative abundance of reads at species level (Askimsbadet).

**Figure 6.**
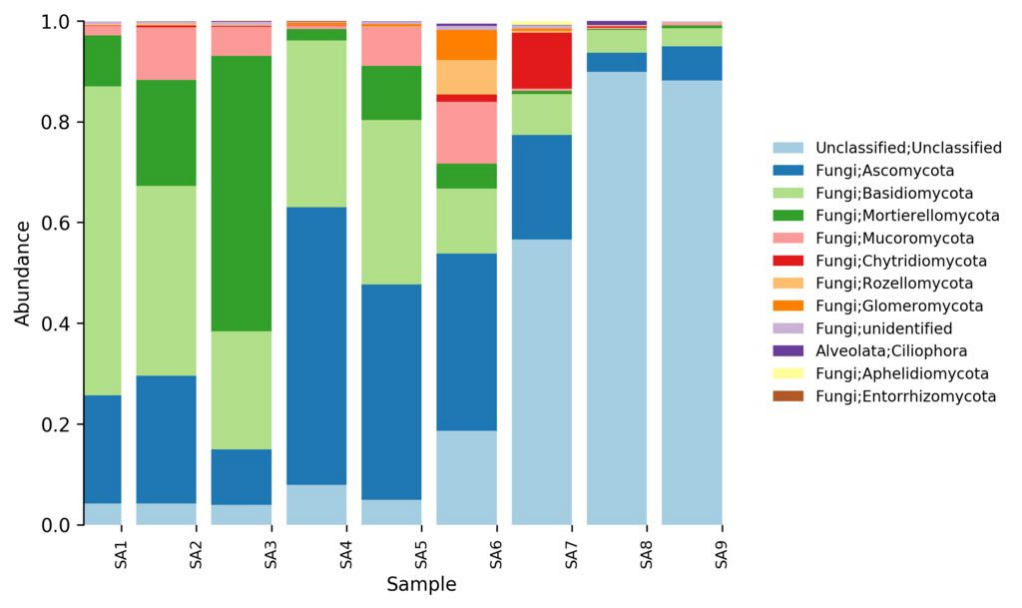
Relative abundance of reads at phylum level (Stora Amundön).

**Figure 7.**
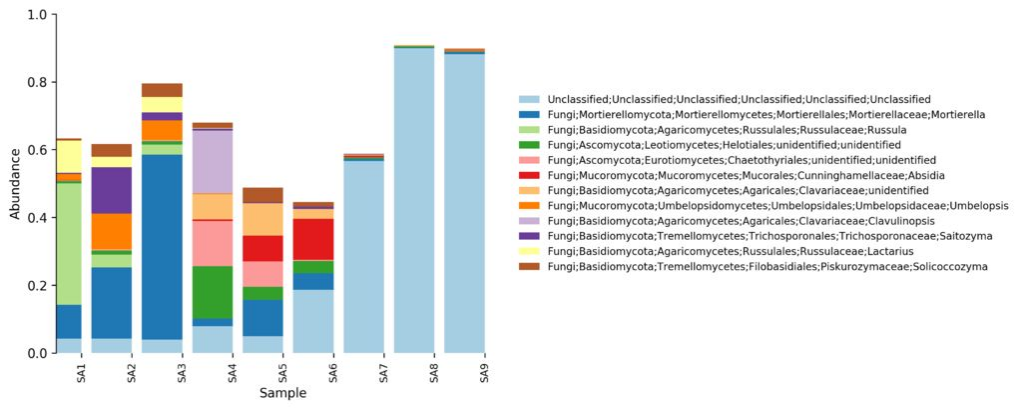
Relative abundance of reads at genus level (Stora Amundön)

**Figure 8.**
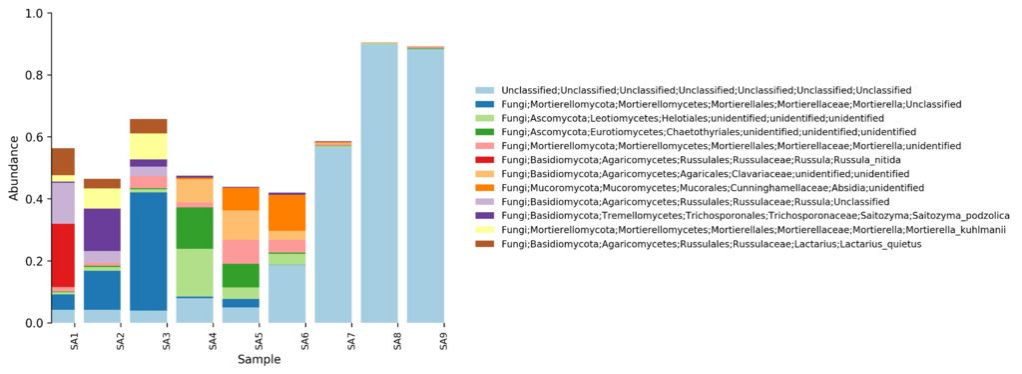
Relative abundance of reads at species level (Stora Amundön).

**Relative abundance tables**: The relative abundances of sequences, including their taxonomic assignment, are visualized in Figs 3, 4, 5, 6, 7, 8. One striking aspect of the relative abundance of sequences is the number of unclassified sequences, >80% at the benthic sites, regardless of the locality or taxonomic level.

**Figure 9.**
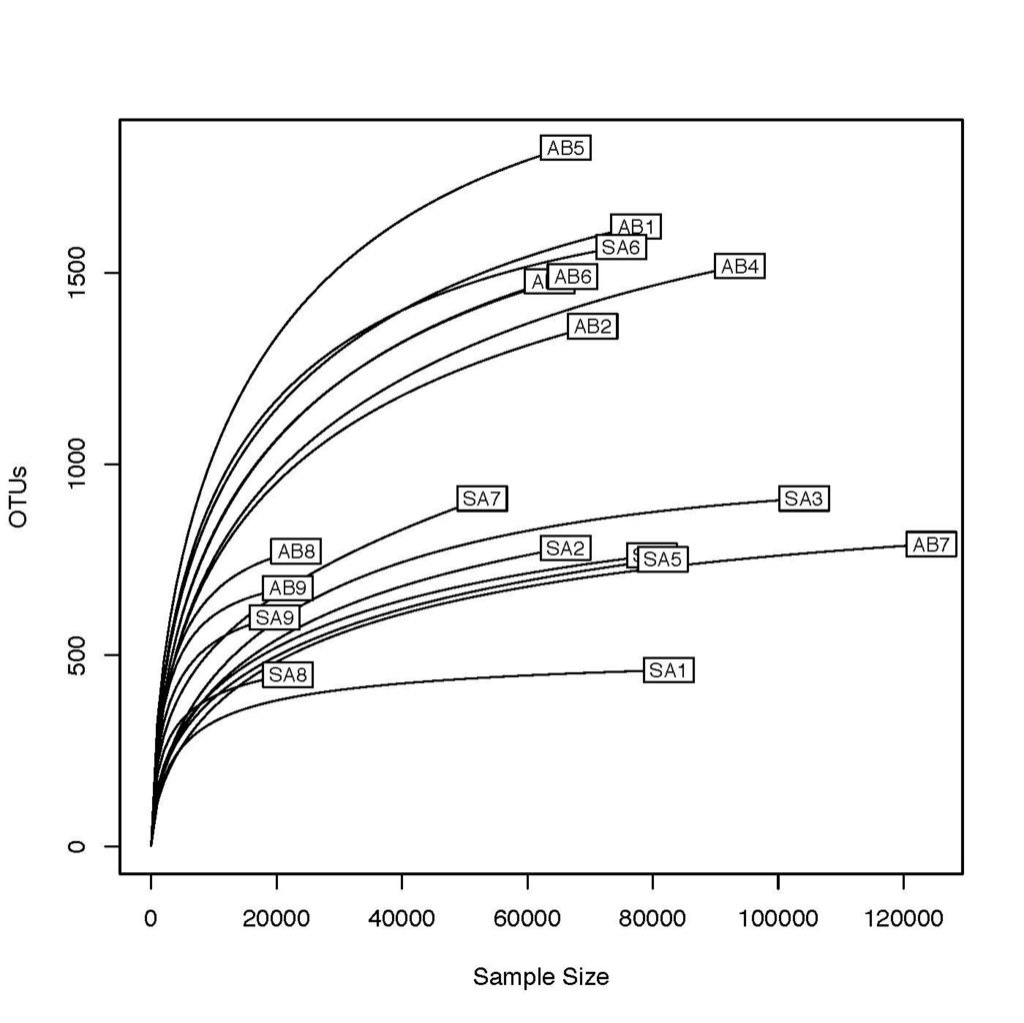
The rarefaction curves represent the means of repeated re-sampling of samples within the transects of Stora Amundön and Askimsbadet, and to some extent species diversity.

**Rarefaction curves**: The rarefaction curves in Fig. 9 represent the means of repeated re-sampling of samples. Levelling off of the slopes indicates a sufficiently large sampling effort. The lowest sequencing depth of both gradients is around 20,000 reads, and is attributable to the ocean sediment samples (SA8, SA9, AB8, and AB9).

**Diversity indices, number of OTUs and reads**: The number of OTUs and reads, as well as diversity indices per site and gradient are summarized in Table 4 and Table 5. The lowest number of reads that each of the tables were rarefied to was taken from SA8 (18,996 reads) and AB9 (22,101 reads), respectively. The number of OTUs recovered is on average lower for the Stora Amundön gradient (744 (285)) than for the Askimsbadet gradient (1232 (399)). The estimate of richness (570 (204)) differs from the true species richness chao1 (722 (266)) by around 150 OTUs in Stora Amundön. In Askimsbadet, the richness (945 (265)) differs from Chao1 (1,214 (406)) by around 270 OTUs. Alpha diversity increases slightly towards the benthic sediments in Stora Amundön (4.23 (0.63)), but it is similar overall. For Askimsbadet it is somewhat constant, except for the intertidal site AB7, which is strikingly lower than the average of the transect (4.8 (0.57)).

**Figure 10.**
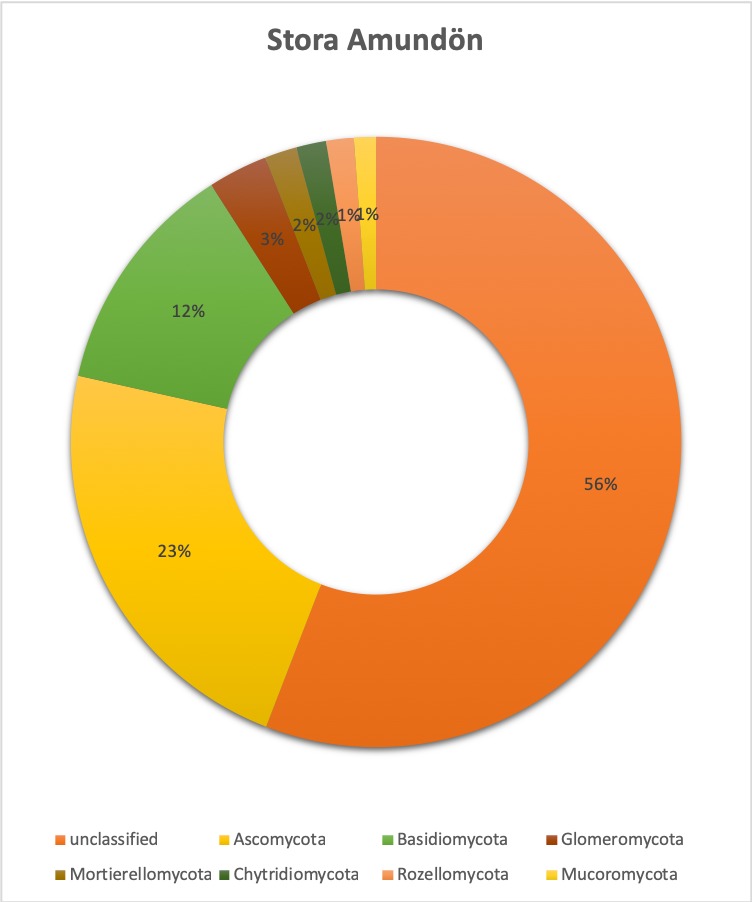
Overall taxonomic coverage (by phylum) within the Stora Amundön transect.

**Figure 11.**
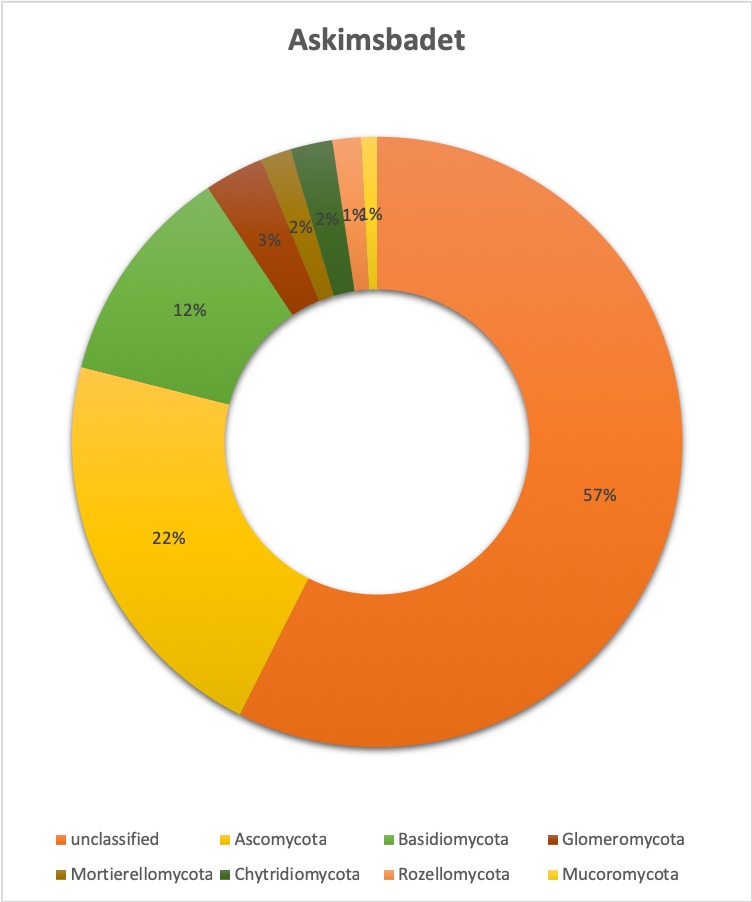
Overall taxonomic coverage (by phylum) within the Askimsbadet transect.

**Taxonomic composition and distribution of OTUs**: We recovered 3470 fungal OTUs from the Stora Amundön transect (Fig. 10, OTU table and taxonomy in Suppl. materials 4, 6). 1872 OTUs (~53%) could not be classified at the phylum level. The remaining 1598 OTUs comprised mainly Dikarya (composed of 21.8% Ascomycota and 12% Basidiomycota) followed by species of Glomeromycota (3.1%), whereas Mortierellomycota, Chytridiomycota, and Rozellomycota were about equally abundant (~1.5%). In the Askimsbadet gradient, 2371 out of 4315 fungal OTUs (~55%) could not be classified to the phylum level (Fig. 11, OTU table and taxonomy in Suppl. materials 3, 5). The Askimsbadet locality gives a similar picture of the remaining 1998 OTUs, namely one dominated by Dikarya (20.6% representing Ascomycota and 11.2% for Basidiomycota), followed by Glomeromycota (3%), and Chytridiomycota (2.1%), whereas Mortierellomycota and Rozellomycota were fairly equally abundant (~1.5%).

**Figure 12.**
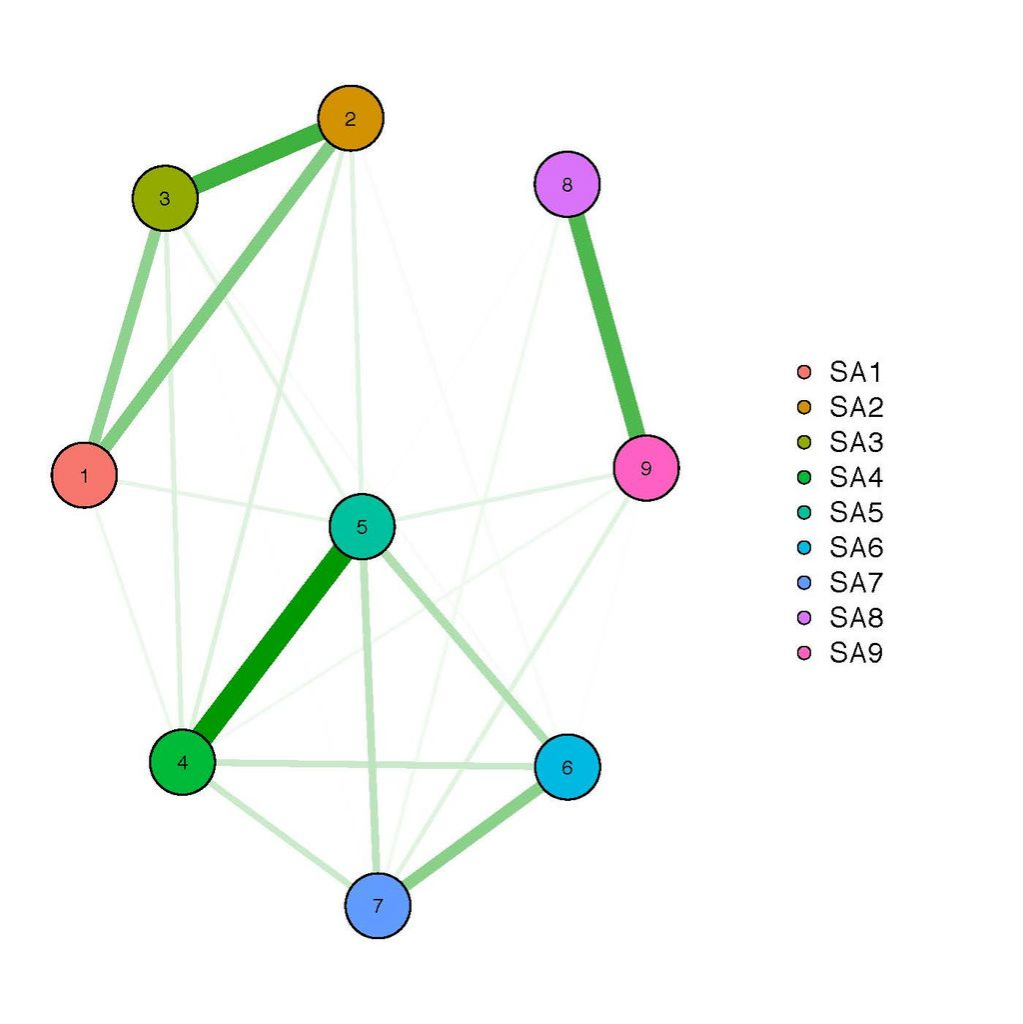
Network graph of beta richness (Stora Amundön).

**Figure 13.**
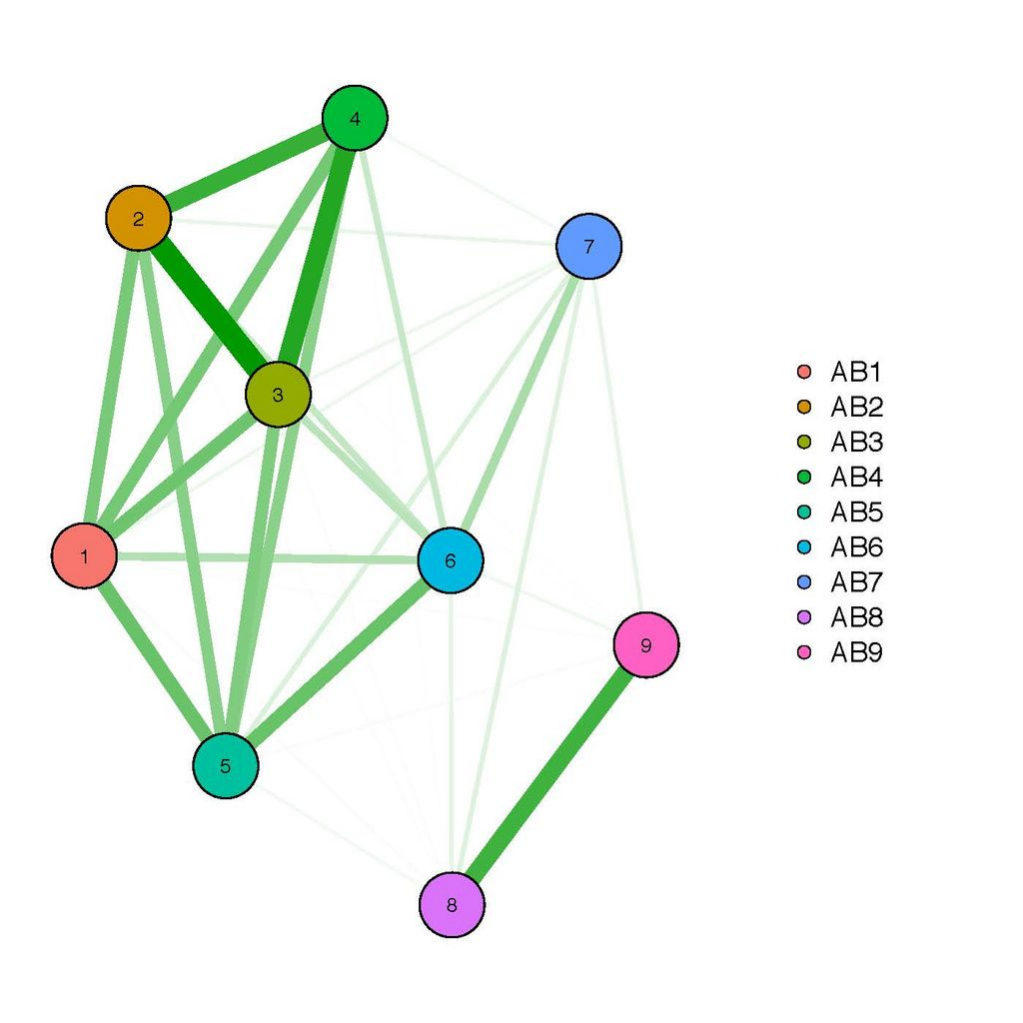
Network graph of beta richness (Askimsbadet).

**Network plot of similar sites**: Thick lines indicate stronger similarities between sites than thinner lines. Beta richness was computed on the basis of the Jaccard index of dissimilarity that is estimated based on the presence/absence-transformed, rarefied OTU tables. The Jaccard index is a popular measurement for beta diversity and proportional species turnover. We could assign the strongest connections to distinct groups representing different environments. For Stora Amundön (Fig. 12) these were: forest group (SA1, SA2, SA3), meadow group (SA4, SA5, SA6, SA7), and ocean (SA8, SA9). For Askimsbadet (Fig. 13) they were: meadow/scattered tree group (AB1, AB2, AB3, AB4), meadow/reed group (AB5, AB6, AB7), and ocean group (AB8, AB9), although AB1 could not be so clearly assigned.

**Figure 14.**
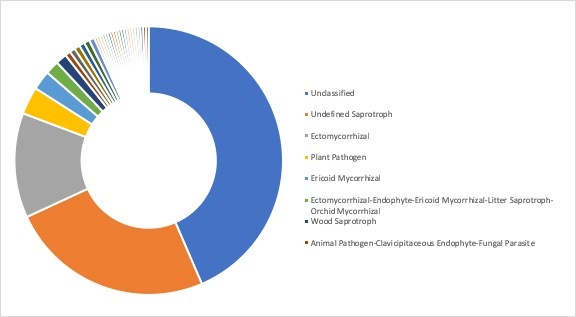
Ecological roles of the fungi recovered from sampling site SA1, representative for the forest group in Stora Amundön (most abundant guilds: 43.5% unclassified, 24.6% undefined saprotrophs, 12.6% ectomycorrhizal, and 3.3% plant pathogens).

**Figure 15.**
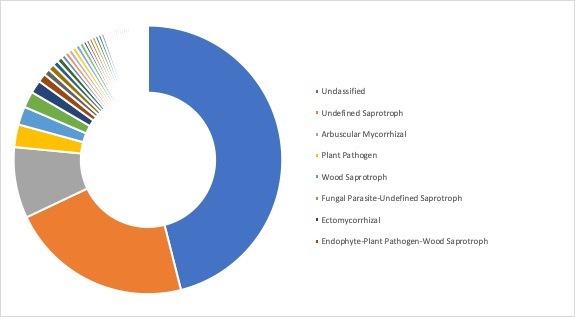
Ecological roles of fungi recovered from sampling site AB2, representative for the meadow/scattered tree group in Askimsbadet (most abundant guilds: 46% unclassified, 21.9% undefined saprotrophs, 8.6% arbuscular mycorrhizal, and 2.7% plant pathogens).

**Figure 16.**
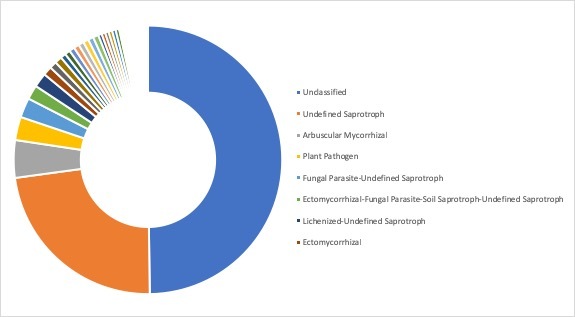
Ecological roles of fungi recovered from sampling site SA5, representative for the meadow group in Stora Amundön (most abundant guilds: 49.8% unclassified, 23.1% undefined saprotrophs, 4.5% arbuscular mycorrhizal, and 2.8% plant pathogens).

**Figure 17.**
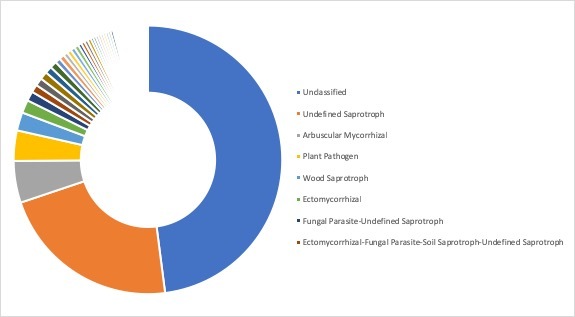
Ecological roles of fungi recovered from sampling site AB5, representative for the meadow/reed group in Askimsbadet (most abundant guilds: 48% unclassified, 21.9% undefined saprotrophs, 5% arbuscular mycorrhizal, and 3.7% plant pathogens).

**Figure 18.**
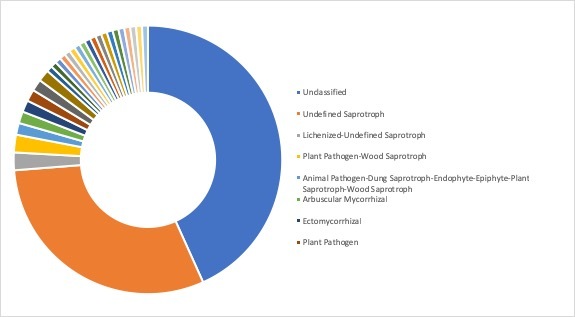
Ecological roles of fungi recovered from sampling site SA9, representative for the ocean group in Stora Amundön (most abundant guilds: 43.3% unclassified, 30.5% undefined saprotrophs, 2.1% lichenized-undefined saprotrophs, and 2.1% plant pathogen-wood saprotrophs).

**Figure 19.**
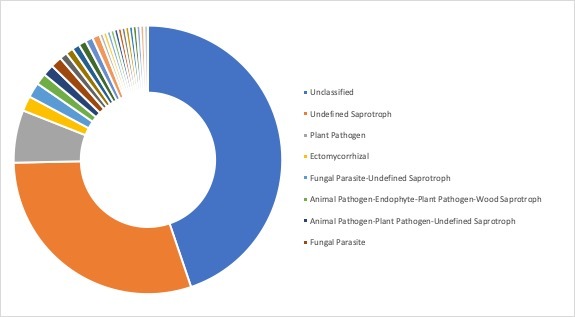
Ecological roles of fungi recovered from sampling site AB9, representative for the ocean group in Askimsbadet (most abundant guilds: 44.8% unclassified, 29.9% undefined saprotrophs, 6.3% plant pathogens, and 1.8% ectomycorrhizal).

**Ecological roles**: Ecological roles of the fungal communities at each site were inferred using FUNGuild (Nguyen et al. 2016) and are visualized in Figs 14, 15, 16, 17, 18 to Fig. 19, with a detailed description given in Suppl. material 1. In each representative group, around 45% of the identified OTUs could not be classified into functional categories by FUNGuild. In total, we were able to assign ~22.5% of the total OTUs to functional guilds.

### Discussion

Recent environmental sequencing studies point to the existence of diverse, but not yet thoroughly understood, communities of fungi recovered from various marine habitats (Rédou et al. 2015, Tisthammer et al. 2016, Picard 2017). In this study, we explored two littoral gradients in Western Sweden that were assessed for their fungal abundances, taxonomic composition, diversity, and ecological roles. Studies using molecular methods to investigate marine fungi in the Swedish west coast are rare, and records of marine fungal taxa have only been described through cultivation approaches (Tibell et al. 2019). Other studies have investigated marine fungi through cultivation of isolates and subsequent molecular analyses in other parts of Scandinavia and the Northern hemisphere. They recovered a more or less similar composition of fungal groups within the ocean with respect to our results, where Dikarya and Chytridiomycota are dominating, and they too reported a significant number of as-yet unclassified OTUs (Rämä et al. 2014, Hassett et al. 2017).

**Soil physicochemical properties**: The physiochemical properties of the soil constitute an important part of ecosystem characterization, and many geochemical processes within the soil lead to changes in ratios of stable isotopes of carbon and nitrogen (Neff et al. 2002, Tiunov 2007). The forest group in the Stora Amundön transect stands out for its relatively higher C/N contents, which could be interpreted as consistent with the amount of organic material available in forests compared to grass landscapes. The soil d^13^C signature can be indicative of the growth of mycorrhizal (~-25‰) or saprotrohic fungi (~-22‰) (Hobbie et al. 1999). The d^15^N fraction indicates how much of the two stable isotopes of nitrogen that was fractionated during nitrogen assimilation and can give a clue to the changes caused by processes such as nitrification and ammonification (Tiunov 2007) and also the trophic level of the organisms in the soil. Bulk tissues of organisms higher up in the trophic chain usually accumulate the heavier ^15^N isotope and vice versa (Griffith 2004). The close-to-zero numbers of the d^15^N measurements in the SA forest sites indicate a relatively higher level of the lighter ^14^N isotope. This suggests that these soils potentially contain organisms lower in the food chain, notably saprotrophs. Some studies suggest a relatively higher d^15^N content when carbon is low, as in mineral soils (Tiunov 2007, Craine et al. 2015), and relatively higher d^15^N values in non-mycorrhizal and arbuscular mycorrhizal plants (Michelsen et al. 1998). Our results follow this pattern.

**Taxonomic composition and primer bias**: One explanation of why mostly members of Dikarya are recovered from environmental sequencing efforts of marine fungi is that they are more readily amplified due to, e.g., methodological shortcomings, including biases in sample preparation and analysis. PCR amplification of the ITS2 metabarcode does not fully reflect the true abundance of species, as the ITS2 region has different copy numbers in different species and organisms due to the tandem repeat nature of the whole rDNA gene cluster (Vilgalys and Hester 1990). Metabarcoding approaches are also sensitive to primer choice as well as PCR biases and thus can only ever be a semi-quantitative approach for environmental sampling efforts. The IT2 barcode, although robust, does not have sufficient phylogenetic signal for higher-level classification, but generally performs well in terms of resolution at the species level (Schoch et al. 2012, Nilsson et al. 2014, Tedersoo et al. 2015). rRNA gene sequences such as the whole or parts of the LSU and/or SSU could be used to build a more robust phylogenetic backbone (Tedersoo et al. 2018).

Both Calvez et al. (2009) and Richards et al. (2015) report results consistent with the assumption that marine environments host numerous unclassified, deeply branching lineages of fungi. The results of the present study support these claims, as more than 80% of our sequences could not be classified even at the phylum level, suggesting that they may belong to new, undescribed fungal lineages or otherwise unsampled (or unsequenced) fungal taxa. Even though we do not know which phyla these unclassified sequences belong to, we can, with relative certainty, place them in the kingdom fungi since we used fungus-specific primers and thorough data processing to scrutinize the sequences for artificial and non-fungal OTUs.

**Species diversity of the samples**: Our diversity estimates should be reasonably accurate as indicated by the rarefaction curves, which are near the asymptote. They indicate a sufficient sampling effort, and a close-to-complete account of the fungal richness, although the curves of the ocean site are considerably shorter as these sites contain a fewer number of reads. To account for differences in copy numbers across fungi, we transformed the read numbers into a binary presence/absence matrix for beta diversity analysis. Evenness as well as the Shannon index are higher in the foreshore and seabed sites of Stora Amundön than in the forest sites, but quite similar to the soil sampled from the meadow landscape. This suggests that the overall diversity in the foreshore and benthic sites is higher than in the forest. Lower evenness numbers would suggest that a few species are relatively more abundant than others, which would have a negative impact on species diversity estimates. In the Askimsbadet gradient, evenness and richness are consistent with the results of the Stora Amundön samples, with the exception of the seashore site AB7, which seems to have the lowest estimate of species diversity. Lower diversity estimates could occur due to the nature of the site itself, but could also be caused by some artefact during DNA extraction from mineral/sandy or very humus-rich soils.

The sampling site groupings in the network plot of site similarities fit the actual habitats the samples were taken from. This suggests that sites from different habitats are indeed more different among each other than within them, diversity-wise. It also indicates the reasonable use of one site as a habitat representative when it comes to the allocation of OTUs into different guilds.

**Limitations and shortcomings**: The occurrence of trophic groups such as ectomycorrhizal fungi within the marine sediment could be potentially explained by DNA from dispersed spores, fragmented fungal hyphae, or perhaps soil washed into the ocean from land. This relic DNA, that is DNA stemming from dead or metabolically inactive cells, can sometimes make up to 40% of ITS sequences recovered from soil (Carini et al. 2017) and is typically hard to discriminate against. Moreover, the seabed we took the benthic sediment samples from was in both transects particularly close to diverse mixed forests, hinting perhaps at natural deposition of fungal propagules. Another possible explanation of the presence of ectomycorrhizal fungi within marine sediments could be a shortcoming in correct guild allocation in FUNGuild – a resource known to be incomplete, just like all other databases covering the fungal kingdom.

**Conclusion**: The present study confirms that there is a disproportionately large number of fungal lineages waiting to be discovered and described, especially in marine environments. This study could be used as a starting point for an estimate and characterization of as-yet undescribed fungal species (probably even phyla) that could potentially be found in this and other temperate zones of the Northern Hemisphere.

Interestingly, we found many “terrestrial” lineages also in the marine samples, showing that aquatic mycology cannot be pursued in isolation from traditional mycology. Similarly, our edaphic data hinted at processes and correlations that we could not fully explain. It seems clear that improved collaboration among scientific disciplines is needed to tackle the explosion of new data emerging from fungal community metabarcoding studies.

Our data show that there are predominantly endophytic, parasitic, and pathogenic fungi in our marine samples, which hints at the presence of interesting and currently poorly understood fungus-driven ecological processes in the marine environment. It is also clear from our results that a very large number of marine fungi are in urgent need of taxonomic study and formal description.

## Supplementary Material

BB413548A740535997E57C7DB397FB0E 10.3897/BDJ.7.e35332.suppl1 Supplementary material 1 Ecological roles of fungal communities Data type: occurences, ecology
Brief description: A detailed description of ecological roles and taxonomy of the fungal communities inferred using FUNGuild.
File: oo_277636.pdf https://binary.pensoft.net/file/277636 Alice Retter, R. Henrik Nilsson, Sarah Bourlat

7A2C868FBB20593C9C8585001BE90181 10.3897/BDJ.7.e35332.suppl2 Supplementary material 2 Raw data statistics of reads Data type: genomic
Brief description: Raw data statistics, showing the total number of bases, reads, GC content (%), AT content (%), the ratio of reads that have phred quality scores of over 20 (%), and the ratio of reads that have phred quality scores of over 30 (%).
File: oo_292782.tsv https://binary.pensoft.net/file/292782 Alice Retter, R. Henrik Nilsson, Sarah Bourlat

7F9806B8CA935E56968955AD20DFCDB5 10.3897/BDJ.7.e35332.suppl3 Supplementary material 3 OTU table Askimsbadet Data type: occurences
Brief description: The OTU table of Askimsbadet, which was scrutinized for non-fungal OTUs.
File: oo_290826.txt https://binary.pensoft.net/file/290826 Alice Retter, R. Henrik Nilsson, Sarah Bourlat

E90AD999186853BDA6AA7835CD60EB3A 10.3897/BDJ.7.e35332.suppl4 Supplementary material 4 OTU table Stora Amundön Data type: occurences
Brief description: The OTU table of Stora Amundön, which was scrutinized for non-fungal OTUs.
File: oo_290828.txt https://binary.pensoft.net/file/290828 Alice Retter, R. Henrik Nilsson, Sarah Bourlat

B0CDF584F67E589A97C216BE8138893C 10.3897/BDJ.7.e35332.suppl5 Supplementary material 5 Taxonomy of Askimsbadet OTUs Data type: taxonomic
Brief description: The taxonomic annotations of the OTUs of Askimsbadet, which were assigned using the UNITE reference database.
File: oo_292780.txt https://binary.pensoft.net/file/292780 Alice Retter, R. Henrik Nilsson, Sarah Bourlat

241132438679540B95DD9CE2680A022A 10.3897/BDJ.7.e35332.suppl6 Supplementary material 6 Taxonomy of Stora Amundön OTUs Data type: taxonomic
Brief description: The taxonomic annotations of the OTUs of Stora Amundön, which were assigned using the UNITE reference database.
File: oo_292781.txt https://binary.pensoft.net/file/292781 Alice Retter, R. Henrik Nilsson, Sarah Bourlat

## Figures and Tables

**Table 1.** Sequence of the forward primer ITS3-Mix2 (Tedersoo et al. 2015) and reverse primers ITS4-cwmix1 and ITS4-cwmix2 (Wurzbacher et al. 2017) for amplification of the fungal ITS2 region. The adapter, spacer, and marker-specific sequence are separated by hyphens. DNA ambiguity symbols follow Cornish-Bowden (1985).

ITS3-Mix2	TCGTCGGCAGCGTCAGATGTGTATAAGAGACAG-AAC-caWCGATGAAGAACGCAg
ITS4-cwmix1	GTCTCGTGGGCTCGGAGATGTGTATAAGAGACAG-AA-TCCTCCGCTTAyTgATAtGc
ITS4-cwmix2	GTCTCGTGGGCTCGGAGATGTGTATAAGAGACAG-AA-TCCTCCGCTTAtTrATAtGc

**Table 2.** Metadata summary of both gradients, Stora Amundön and Askimsbadet, with sampling sites 1-9. Here, C is the mass fraction of carbon, N is the mass fraction of nitrogen, d^13^C is the isotopic ratio of the ^13^C to ^12^C isotopes, d^15^N is the isotopic ratio of the ^15^N to ^14^N isotopes, FC is the isotopic amount of the fraction ^13^C/(^12^C+^13^C), FN is the isotopic amount of the fraction ^15^N/(^14^N+^15^N), and C/N is the ratio of carbon to nitrogen. Underlined numbers were outside of the calibration range.

**Sampling site**	**ω_N_**/%	**d^15^N**/‰	**F_N_**/%	**ω_C_**/%	**d^13^C**/‰	**F_C_**/%	**C/N**	**Conductivity (μS/m)**	**PH**
**AB1**	0.35	5.04	0.3681	4.75	-28.98	1.079377	13.6	121.5	5.97
**AB2**	0.50	5.01	0.3681	7.87	-28.96	1.079399	15.7	130.0	6.13
**AB3**	0.30	4.99	0.3681	4.39	-28.70	1.079685	14.6	144.5	5.30
**AB4**	0.51	5.95	0.3685	7.44	-28.58	1.079817	14.6	170.5	5.80
**AB5**	0.57	6.47	0.3687	7.32	-27.61	1.080883	12.8	207.0	6.67
**AB6**	0.40	6.80	0.3688	6.23	-19.07	1.090273	15.6	276.5	7.40
**AB7**	0.05	7.50	0.3690	0.79	-16.47	1.093131	15.8	3830.0	6.79
**AB8**	0.07	4.85	0.3681	0.90	-14.73	1.095044	12.9	2810.0	7.92
**AB9**	0.05	4.92	0.3681	0.72	-14.70	1.095077	14.4	2315.0	7.94
**SA1**	2.04	-0.06	0.3663	44.68	-28.50	1.079905	21.9	220.0	4.32
**SA2**	1.39	-0.01	0.3663	32.59	-29.48	1.078827	23.4	257.5	4.02
**SA3**	1.54	0.25	0.3664	35.69	-28.57	1.079828	23.2	145.5	3.98
**SA4**	0.36	3.77	0.3677	5.17	-27.37	1.081147	14.4	133.5	5.28
**SA5**	0.56	3.96	0.3677	8.19	-27.40	1.081114	14.6	150.0	5.51
**SA6**	0.43	3.98	0.3678	6.07	-28.87	1.079498	14.1	271.5	6.68
**SA7**	0.01	5.45	0.3683	0.08	-21.08	1.088063	8,0	398.0	7.14
**SA8**	0.02	4.82	0.3681	0.28	-12.34	1.097671	14.0	5755.0	8.43
**SA9**	0.03	4.65	0.3680	0.87	-5.75	1.104914	29.0	5015.0	8.42

**Table 3.** Number of reads after merging, primer trimming, and quality filtering of reads shorter than 260 bases and/or with an expected error rate of >0.5%. The number of discarded chimeric sequences is also indicated. Number of reads after each processing step in micca (Albanese et al. 2015).

	**Stora Amundön**	**Askimsbadet**
Reads after merging	687600	789758
Reads after primer trimming	686338	788012
Reads after filtering	673711	779899
Chimeric sequences discarded	546	729

**Table 4.** Number of sequences, OTUs, and basic diversity indices of Stora Amundön (SA).

	**SA1**	**SA2**	**SA3**	**SA4**	**SA5**	**SA6**	**SA7**	**SA8**	**SA9**
**Reads**	82,577	66,043	104,082	79,767	81,595	77,016	52,967	18,996	19,139
**OTUs**	447	746	865	693	688	1,371	883	426	574
**Richness**	373.94	516.77	568.23	491.13	472.64	1,070.33	637.80	426.00	573.55
**Evenness**	0.59	0.62	0.55	0.65	0.63	0.76	0.73	0.74	0.76
**Chao1**	424.93	694.98	770.26	699.77	622.27	1,298.00	931.90	451.24	602.02
**Shannon**	3.49	3.83	3.50	4.03	3.91	5.27	4.73	4.51	4.81

**Table 5.** Number of sequences, OTUs, and basic diversity indices of Askimsbadet (AB).

	**AB1**	**AB2**	**AB3**	**AB4**	**AB5**	**AB6**	**AB7**	**AB8**	**AB9**
**Reads**	77,433	70,132	63,519	93,882	65,999	64,737	124,351	23,485	22,101
**OTUs**	1561	1310	1411	1458	1742	1425	771	752	655
**Richness**	1153.9	963.35	1069.02	988.68	1339.56	1087.3	496.72	747.51	655
**Evenness**	0.71	0.7	0.68	0.67	0.76	0.72	0.55	0.76	0.76
**Chao1**	1483.51	1279.37	1548.71	1264.35	1752.95	1469.3	674.34	782.86	670.45
**Shannon**	5.00	4.82	4.78	4.64	5.50	5.05	3.43	5.05	4.92
