# Supplementary material for: Exploring the taxonomic composition of two fungal communities on the Swedish west coast through metabarcoding
Source: Biodivers Data J. 2019 Sep 4;7:e35332. doi: 10.3897/BDJ.7.e35332 (PMC6739426; doi:10.3897/BDJ.7.e35332)
Supplement: Supplementary material 1 — Ecological roles of fungal communities [file bdj-07-e35332-s001.pdf]

In the forest sites of Stora Amundön (*Fig. 14*), the classifiable OTUs are mainly allocated into guilds of undefined saprotrophs (genera such as *Mortierella*, *Umbelopsis*, *Xenasmatella*, *Cladophialophora*, *Arachnopeziza*, *Peniophorella*, *Exophiala*, *Filobasidium*, *Mucor*, and *Botryobasidium*, and the orders Eurotiales and Hypocreales (Pezizomycotina), Saccharomycetales (Saccharomycotina), and Mucorales (Mucormycotina)), ectomycorrhizal fungi (genera such as *Cortinarius*, *Russula*, *Amanita*, *Pseudotomentella*, *Elaphomyces*, *Lactarius*, *Tricholoma*, *Tuber*, and *Cenococcum*), and plant pathogens (*Coccomyces.*, *Venturia*, *Adisciso*, *Kochiomyces*, *Verticillium*, and *Rhizophydium*). These account for around 41 % of the total number of guilds, as the rest are either very small or unclassifiable. The guilds with the highest number of sequences are mostly ectomycorrhizal that grow symbiotrophically.

In the meadow/scattered tree sites (*Fig. 15*), the classifiable OTUs are mainly allocated into guilds of undefined saprotrophs (genera such as *Mortierella*, *Umbelopsis*, *Tetracladium*, *Luellia Rhizophlyctis*, *Marasmiellus*, *Apodus*, *Exophiala*, *Dactylella*, *Ophiosphaerella*, and *Coprinopsis*, and orders such as Eurotiales and Hypocreales (Pezizomycotina), Saccharomycetales (Saccharomycotina), and Mucorales (Mucormycotina)), arbuscular mycorrhizal fungi (genera such as *Claroideoglomus*, *Scutellospora*, *Archaeospora*, *Rhizophagus*, and *Acaulospora* as well as three orders of Glomeromycota (Archaeosporales, Paraglomerales, and Glomerales)) and plant pathogens (genera such as *Rhizophydium* , *Entorrhiza*, *Drechslera*, *Ustilago*, *Waitea*, *Venturia*, *Itersonilia*, and *Pseudopeziza.*). These account for around 33 % of the total number of guilds, and the rest are either very small or unclassifiable. The guilds with the highest number of sequences are a diverse selection of saprotrophic, lichenized, ectomycorrhizal, fungal parasites, and/or plant pathogens that show a variety of saprotrophic, pathotrophic, and/or symbiotrophic associations.

In the meadow sites (*Fig. 16*), the classifiable OTUs are mainly allocated into guilds of undefined saprotrophs (genera such as *Mortierella*, *Umbelopsis*, *Geoglossum*, *Tetracladium*, *Clavulinopsis*, *Pholiota*, *Gymnostellatospora*, *Glutinoglossum*, *Leohumicola*, *Brevicellicium*, and *Clavaria*, and orders such as Eurotiales and Hypocreales (Pezizomycotina), Saccharomycetales (Saccharomycotina), and Mucorales (Mucormycotina)), arbuscular mycorrhizal fungi (genera such as *Claroideoglomus* and *Rhizophagus* as well as two orders of Glomeromycota (Gigasporales and Glomerales)), and plant pathogens (genera such as *Entorrhiza*, *Devriesia*, *Drechslera*, *Ustilago*, *Thanatephorus*, *Clonostachys*, *Itersonilia*, *Plectosphaerella*, and *Septoria*). These account for around 30 % of the total number of guilds, and the rest are either very small or unclassifiable. The guilds with the highest number of sequences are lichenized and/or saprotrophs that grow saprotrophically or saprotrophic-symbiotrophically.

In the meadow/reed sites (*Fig. 17*), the classifiable OTUs are mainly allocated into guilds of undefined saprotrophs (genera such as *Mortierella*, *Preussia*, *Exophiala*, *Ophiosphaerella*, *Rhizophlyctis*, *Tetracladium*, *Clitopilus*, *Arthrobotrys*, *Coprinopsis*, *Lycoperdon*, and *Mucor*, and orders such as Eurotiales and Hypocreales (Pezizomycotina), Saccharomycetales (Saccharomycotina), and Mucorales (Mucormycotina)), arbuscular mycorrhizal fungi (genera such as *Claroideoglomus*, *Diversispora* , and *Scutellospora* as well as the order Paraglomerales and five orders of Glomeromycota (Paraglomerales, Diversisporales, Archaeosporales, Gigasporales, and Glomerales)), and plant pathogens (genera such as *Ustilago*, *Urocystis*, *Plectosphaerella* , *Neonectria*, *Clonostachys*, *Drechslera*, *Rhizophydium*, *Volutella*, and *Itersonilia*). These account for around 30 % of the total number of guilds, and the rest are either very small or unclassifiable. The guilds with the highest number of sequences are saprotrophs or plant and animal pathogens that grow pathotrophically and/or saprotrophically.

In the Stora Amundön transect, the classifiable OTUs of the ocean group (*Fig. 18*) are mainly allocated into guilds of undefined saprotrophs (genera such as *Hypholoma*, *Pseudeurotium*, *Mortierella*, *Umbelopsis*, *Paraphaeosphaeria*, *Glutinoglossum*, *Lycoperdon*, *Teichospora*, *Sarocladium*, *Exophiala*, *Leohumicola*, and *Mucor*, and orders such as Eurotiales and Hypocreales (Pezizomycotina), Saccharomycetales (Saccharomycotina), Mucorales (Mucormycotina)), lichenized-undefined saprotrophs (Clavariaceae), and plant pathogen-wood saprotrophs (*Phoma*, *Stemphylium*, and *Fusarium*). These account for about 35 % of the total number of guilds, and the rest are either very small or unclassifiable. In both transcects, guilds with the highest number of sequences are endophyte plant-, and/or animal pathogens that grow pathotrophically or pathotroph-saprotroph-symbiotrophically.In the Askimsbadet transect, the classifiable OTUs of the ocean group (*Fig. 19*) are mainly allocated into guilds of undefined saprotrophs (genera such as *Hypholoma*, *Pseudeurotium*, *Mortierella*, *Umbelopsis*, *Lycoperdon*, *Teichospora*, *Geoglossum*, *Leohumicola*, *Clitopilus*, *Exophiala*, and *Mucor*, and orders such as Eurotiales and Hypocreales (Pezizomycotina), Saccharomycetales (Saccharomycotina), and Mucorales (Mucormycotina)), plant pathogens (*Urocystis*, *Ustilago*, *Paradendryphiella*, *Leptosphaeria*, *Volutella*, and *Microbotryum*), and ectomycorrhizal fungi (*Byssocorticium*, *Chloridium*, *Suillus*, and *Amphinema*). These account for about 38 % of the total number of guilds, and the rest are either very small or unclassifiable.
